# Implications of personality and parental education on healthy lifestyles among adolescents

**DOI:** 10.1038/s41598-020-64850-3

**Published:** 2020-05-13

**Authors:** Aina M Yañez, Miquel Bennasar-Veny, Alfonso Leiva, Mauro García-Toro

**Affiliations:** 1 Department of Nursing and Physiotherapy, University of the Balearic Islands, Palma de Mallorca, Illes Balears Spain; 2 Research Group on Global Health & Human Development, Balearic Islands University, Palma, Illes Balears Spain; 3 Primary Care Research Unit of Mallorca, Balearic Islands Health Service, Instituto de Investigación Sanitaria Illes Balears, Palma, Illes Balears Spain; 4 Department of Medicine, University of the Balearic Islands, Palma, Illes Balears Spain

**Keywords:** Public health, Psychiatric disorders, Risk factors

## Abstract

Several studies have shown an association between personality and health status. The aim of this study was to evaluate the association between personality traits, parental education and health-related lifestyles in a cohort of Spanish adolescents. This is a longitudinal study with a source population of 1,123 third-year students (aged 14–15) in secondary schools in Spain. At the baseline evaluation sociodemographic variables, parental education and personality (Big Five Questionnaire for Children) were collected. At 18 months of follow-up health related lifestyles, including adherence to a healthy diet (KidMed index), tobacco and alcohol consumption, physical exercise, sleep problems and recreative screen and social network time were collected. A total of 824 adolescents (73.4%) completed the 18 months assessment and 695 (84.3%) presented valid data. Higher conscientiousness was associated to a lower risk for non-adherence to Mediterranean diet (OR = 0.7, 95% CIs=0.5–0.9), tobacco (OR = 0.5, 95% CIs=0.3–0.7) and alcohol consumption (OR = 0.6, 95% CIs=0.5–0.8), excessive use of screens (OR = 0.7, 95% CIs=0.5–0.9) and social network sites (OR = 0.7, 95% CIs=0.5–0.8). Higher levels of extraversion was significantly related to a lower risk of physical inactivity (OR = 0.7, 95% CIs=0.6–0.9), but they are at a higher risk of low adherence to Mediterranean diet (OR = 1.3, 95% CIs=1.0–1.7), tobacco (OR = 2.7, 95% CIs=1.7–4.3) and alcohol consumption (OR = 1.9, 95% CIs=1.5–2.4) and excessive use of social network sites (OR = 1.6, 95% CIs=1.3–1.9). High levels of emotional instability were associated with tobacco consumption (OR = 1.5, 95% CIs=1.0–2.2) and sleep problems (OR = 2.0, 95% CIs=1.5–2.7). Finally, we found an association with lower parental education and adolescents’ low adherence to Mediterranean diet (OR = 1.6, 95% CIs=1.0–2.4) and sleep problems (OR = 1.8, 95% CIs=1.0–3.0). Cluster analysis of health-related behaviours indicated the presence of two different clusters (unhealthy and healthy adolescents) that were associated with personality traits. Conscientiousness, extraversion, emotional instability and parental education are independent factors associated with the acquisition of adolescent healthy lifestyles.

## Introduction

Acquisition of healthy lifestyles during adolescence is critical for the prevention of most non-communicable diseases (hypertension, type 2 diabetes, cardiovascular diseases, and mental illnesses). These diseases commonly occur in adulthood and could be prevented by adopting healthy lifestyles^1^. In addition, those conditions entail high financial and social costs for the society^2^.

Numerous studies have shown an association between personality and health status^3,4^. The behavioural mediation model postulates that the observed association between personality and health would be the result of adopted healthy lifestyles (e.g., healthy diet, physical exercise, etc.), along with the avoidance of behaviours that could be harmful to health (e.g., tobacco, alcohol, etc.). The association between personality and healthy lifestyles begins in early stages of life^5^ and promoting healthy lifestyles among children or adolescents reinforce the adoption of healthy habits and could delay or prevent chronic illnesses in the future.

The Big Five is a theory of personality traits that identifies five distinct factors as central to personality: extraversion, agreeableness, conscientiousness, openness, and emotional instability (neuroticism). However, is still controversial whether personality traits are stable over time^6–8^.

High conscientiousness scores have been related with healthier lifestyles in adults^9–12^. A literature review^13^ indicated that conscientiousness trait in adults predict health status throughout life and is also associated with a lower risk of mortality. Recent research suggests that lower conscientiousness levels in childhood are associated with a lower health status (measured in terms of blood pressure, glucose level, lipid profile, body mass index, and medication consumption) in the adulthood. These associations were maintained even after controlling for conscientiousness in adult life^14^. These results indicate that the association between conscientiousness and mortality could be mediated by lifetime health-related behaviours^15^. The mechanisms involved constitute an important field of research, given that lifestyles are potentially modifiable, especially if interventions are performed early.

Promoting healthy lifestyles should have an important impact in the prevention of diseases. All lifestyles factors are potentially modifiable and there is a robust evidence that interventions to promote healthy lifestyles are effective in the prevention of diabetes^16^, hypertension^17^ and cardiovascular disease^18^.

Southern European countries have the highest rate of child obesity in Europe^19^, one possible cause is the loss of the traditional Mediterranean diet in child and adolescents. The Mediterranean diet is considered one of the healthiest dietary patterns that contributes to the prevention of cardiovascular diseases, hypertension, diabetes, and cancer^20,21^ and could have a protective effect on the incidence of depression in young adults^22,23^. Despite all these benefits, in general and especially among children and adolescents, there has been a progressive abandonment of the food pattern based on the Mediterranean diet in our environment^24,25^.

Regular physical activity entails benefits that contribute to the well-being of individuals with respect to physical, mental and social aspects. Performing physical exercise at least 90 minutes a week reduces all causes of death, including all types of cancer, and there is an increase in life expectancy^26^. In addition, high levels of physical exercise in childhood and adolescence predicts high levels of physical exercise in adulthood^27,28^.

Adolescents who are sleep deprived have greater intolerance to stress, emotional dysregulation, behavioural disorders, depression, and other emotional disorders^29–31^. Different longitudinal studies have indicated that lack of sleep, emotional dysregulation, and depressive symptoms interact with each other simultaneously as causes and consequences^29,30^. In this way, emotional instability could favour emotional dysregulation responses, increasing cognitive activity and inhibiting sleep. In turn, sleep deprivation would alter emotional processing and, therefore, its regulation^30,32^.

There is a lack of robust evidence on the association between personality and lifestyles related to health in a large sample of adolescents. The aim of the present study was to assess the association between personality traits, parental education, and health-related lifestyles among south European adolescents (aged 14 to 15 years). We hypothesized that high levels of conscientiousness and high parental education would be related to healthier lifestyles among adolescents.

## Results

Table 1 shows the lifestyles of adolescents included in the study by sex. The mean age at the baseline evaluation was 14.5 (SD 0.66) years and 57% of the sample was composed of girls. Girls obtained significantly higher scores in agreeableness, conscientiousness, and emotional instability (*p* < 0.05), whereas the boys obtained higher scores in openness (*p* < 0.01) (Table 2).

**Table 1:** Health related lifestyles in adolescents by sex.

	Total Sample n (%) / Mean (SD) n = 695	Boys n (%) / Mean (SD) n = 297	Girls n (%) / Mean (SD) n = 398	p-value
Age	16.0 (0.66)	16.0 (0.7)	16.0 (0.6)	0.274
Physical activity (weekly)	565 (81.3%)	252 (84.8%)	313 (78.6%)	0.041
Optimal Mediterranean Diet adherence	177 (25.5%)	69 (23.2%)	108 (27.1%)	0.203
Tobacco consumption (weekly or daily)	61 (8.8%)	24 (8.1%)	37 (9.4%)	0.621
Alcohol consumption (weekly or daily)	138 (19.9%)	65 (22%)	73 (18.3%)	0.236
Sleep problems one or more nights/week	210 (30.2%)	68 (23%)	142 (35.9%)	<0.001
Every night	58 (8.3%)	14 (4.7%)	44 (11.0%)	
Sleep time (hours)/ weekday	7.6 (1.0)	7.7 (1.1)	7.4 (0.9)	<0.001
Sleep time <8 hours / weekday	393 (56.5%)	148 (49.8%)	245 (61.4%)	<0.001
TV time (hours)/ weekday	1.9 (1.8)	2 (1.9)	1.9 (1.6)	0.554
Screen time (hours)/weekday	5.3 (5.0)	4.5 (4.3)	5.9 (5.3)	<0.001
Social Network time (hours)/weekday	5.4 (6.0)	4.9 (6.3)	5.7 (5.9)	0.050

**Table 2:** Personality and family educational levels by sex.

	Total sample n (%) / Mean (SD n = 695	Boys n (%) / Mean (SD) n = 297	Girls n (%) / Mean (SD) n = 398	p-valor
Openness	25.0 (3.2)	25.9 (3.3)	24.4 (3.0)	<0.001
Conscientiousness	68.7 (9.7)	67.7 (9.6)	69.4 (9.7)	0.022
Extraversion	39.6 (5.1)	39.4 (5.2)	39.7 (5.1)	0.542
Agreeableness	37.7 (4.8)	37.0 (4.8)	38.3 (4.7)	<0.001
Emotional instability	26.3 (7.2)	24.1 (6.3)	27.9 (7.4)	<0.001
Mother’s education (n = 680)				
Less than primary	19 (2.8%)	4 (1.4%)	15 (3.8%)	0.089
Only Primary	175 (25.7%)	73 (25.3%)	102 (26.6%)	
Secondary	337 (49.6%)	138 (47.9%)	199 (50.8%)	
University	149 (21.9%)	73 (25.3%)	76 (19.4%)	
Father’s education (n = 671)				
Less than primary	27 (4.0%)	10 (3.5)	17 (4.5%)	0.219
Only Primary	223 (33.2%)	85 (29.4%)	138 (36.1%)	
Secondary	332 (49.5%)	155 (53.6%)	177 (46.3%)	
University				
	89 (13.3%)	39 (13.5%)	50 (13.1%)	

### Diet

Among the participants, 177 (25.6%) consumed an optimal Mediterranean diet, however 116 (16.7%) adolescents adhere poorly to the Mediterranean diet. There were no statistically significant differences between boys and girls (Table 1).

The students with low adherence to the Mediterranean diet showed lower levels of conscientiousness and higher levels of extraversion. The other personality traits had no statistically significant differences between those who showed good adherence to the Mediterranean diet and those who did not. The students whose parents had higher levels of education exhibited better adherence to the Mediterranean diet than those students whose parents had lower levels of education (p < 0.01).

**Figure 1:**
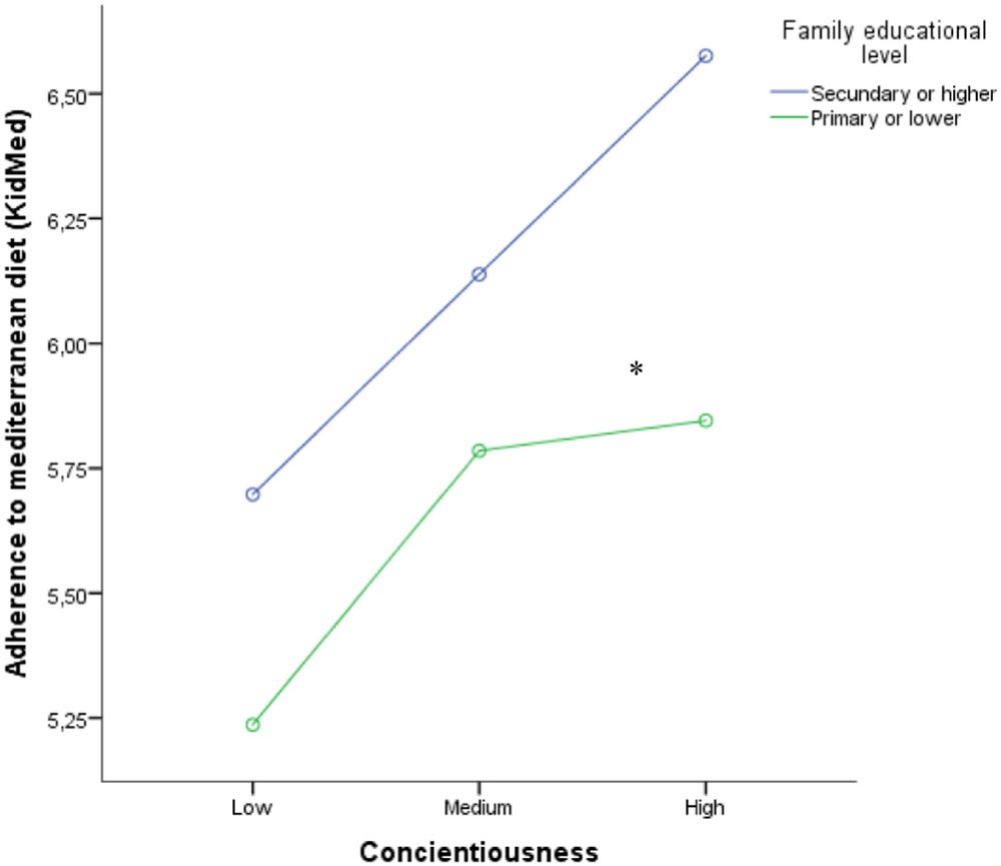
Adherence to Mediterranean Diet by family educational level and conscientiousness among adolescents. *p < 0.01 for both variables (conscientiousness and family educational level) adjusted by age and sex (General Linear Model).

The multivariate logistic regression model indicated that conscientiousness, extraversion, and family educational levels were associated with adherence to the Mediterranean diet, adjusted for age and sex (Table 3). For the general linear model, we considered adherence to the Mediterranean diet as a continuous dependent variable, the level of conscientiousness in terciles (high, medium, and low), and parental educational level as independent variables. In line with the previous analysis, we observed that the increase in the levels of conscientiousness corresponded to a linear increase in the levels of adherence to the Mediterranean diet (*p* < 0.05), and that higher levels of family education were associated with greater adherence to the Mediterranean diet (Fig. 1).

**Table 3:** Association between personality, family educational level and each health-related lifestyles in adolescents.

Variables	Mediterranean Diet (Low Adherence) aOR (IC95%)	No Physical Activity (weekly) aOR (IC95%)	Tobacco consumption (weekly) aOR (IC95%)	Alcohol consumption (weekly) aOR (IC95%)	Sleeping problems (weekly) aOR (IC95%)	Screen time (>2 hours/day) aOR (IC95%)	Social Network use (> 2 hours/day) aOR (IC95%)
Sex (boys vs girls)	1.2 (0.7–1.9)	1.7 (1.1–2.6)*	1.0 (0.5–2.1)	0.6 (0.4–0.9)*	1.5 (0.7–2.9)	1.1 (0.7–1.7)	2.0 (1.4–2.9)*
Low family educational level	1.6 (1.0–2.4)*	1.4 (0.9–2.0)	1.0 (0.5–2.0)	1.3 (0.9–1.9)	1.8 (1.0–3.0)*	1.3 (0.9–2.0)	1.1 (0.8–1.6)
Conscientiousness	0.7 (0.5–0.9)*	0.9 (0.7–1.1)	0.5 (0.3–0.7)*	0.6 (0.5–0.8)*	1.1 (0.8–1.5)	0.7 (0.5–0.9)	0.7 (0.5–0.8)*
Openness	1.0 (0.8–1.3)	1.1 (0.9–1.4)	1.0 (0.7–1.4)	0.7 (0.6–0.8)*	0.9 (0.7–1.3)	0.9 (0.7–1.2)	0.8 (0.7–1.0)
Extraversion	1.3 (1.0–1.7)*	0.7 (0.6–0.9)*	2.7 (1.7–4.3)*	1.9 (1.5–2.4)*	0.9 (0.7–1.3)	1.0 (0.8–1.3)	1.6 (1.3–1.9)*
Agreeableness	0.9 (0.7–1.1)	1.0 (0.8–1.3)	1.0 (0.6–1.5)	1.0 (0.8–1.3)	1.1 (0.8–1.6)	1.1 (0.8–1.4)	1.0 (0.8–1.3)
Emotional instability	1.0 (0.8–1.2)	0.9 (0.8–1.2)	1.5 (1.0–2.2)*	1.3 (1.0–1.6)*	2.0 (1.5–2.7)*	1.1 (0.9–1.3)	1.1 (0.9–1.3)
	Cohen’s d	Cohen’s d	Cohen’s d	Cohen’s d	Cohen’s d	Cohen’s d	Cohen’s d
Conscientiousness	0.259	−0.030	0.539	0.325	0.129	0.328	0.215
Openness	−0.054	−0.060	−0.169	0.154	0.141	0.020	0.136
Extraversion	−0.094	0.370	−0.457	−0.321	0.146	−0.084	−0.274
Agreeableness	0.163	0.075	0.060	0.0152	0.153	0.013	−0.022
Emotional instability	−0.009	0.049	−0.458	−0.114	−0.628	−0.126	−0.246

*p < 0.05; aOR: adjusted Odd Ratio from 7 logistic regression (adjusted by age, sex, personality traits and educational level).

### Physical activity

Boys were more likely to participate in sports activities, 85% of the boys and 79% of the girls performed physical activities at least once a week (*p* < 0.05). Extraversion levels were higher among adolescents who exercised at least once a week (z-score: 40.0 *vs*. 38.1; *p* < 0.001), and there were not differences in agreeableness, conscientiousness, openness, and emotional instability. The logistic regression model, adjusted for age and sex, indicated that high levels of extraversion maintained independent significant association with the usual performance of physical exercise (Table 3).

### Tobacco

Among all the students, 8.8% reported that they were regular smokers (at least once a week) (Table 1). Smoking was associated with lower levels of conscientiousness and higher levels of extraversion and emotional instability. In the multivariate analysis, these associations remained similar (Table 3).

### Alcohol

One in five adolescents reported that they consumed alcohol weekly (Table 1). The bivariate analysis indicated that weekly alcohol consumption was associated with lower conscientiousness, openness, and higher extraversion and emotional instability. In the logistic regression analysis these associations were maintained (Table 3).

### Sleep

Four out of ten students reported that they had sleep problems at least once a week (Table 1). This proportion was higher among girls than among boys (47% *vs*. 28%; *p* < 0.001). In the bivariate analysis, suffering from sleep problems was associated with greater emotional instability and lower levels of parental education (*p* < 0.05). The multivariate analysis indicated that these associations were preserved, except for sex, which had no statistical significance.

### Screen time and social network sites

On average, on school days, the adolescents used the screen 5.3 hours per day, and social networks 5.4 hours per day. In both cases, the use was higher among girls than among boys (Table 1). The logistic regression analysis indicated that lower levels of conscientiousness were associated with excessive use of screens and social networks. Higher levels of extraversion and the female sex were also associated with excessive use of social networks.

### Cluster analysis of health-related lifestyles

**Figure 2:**
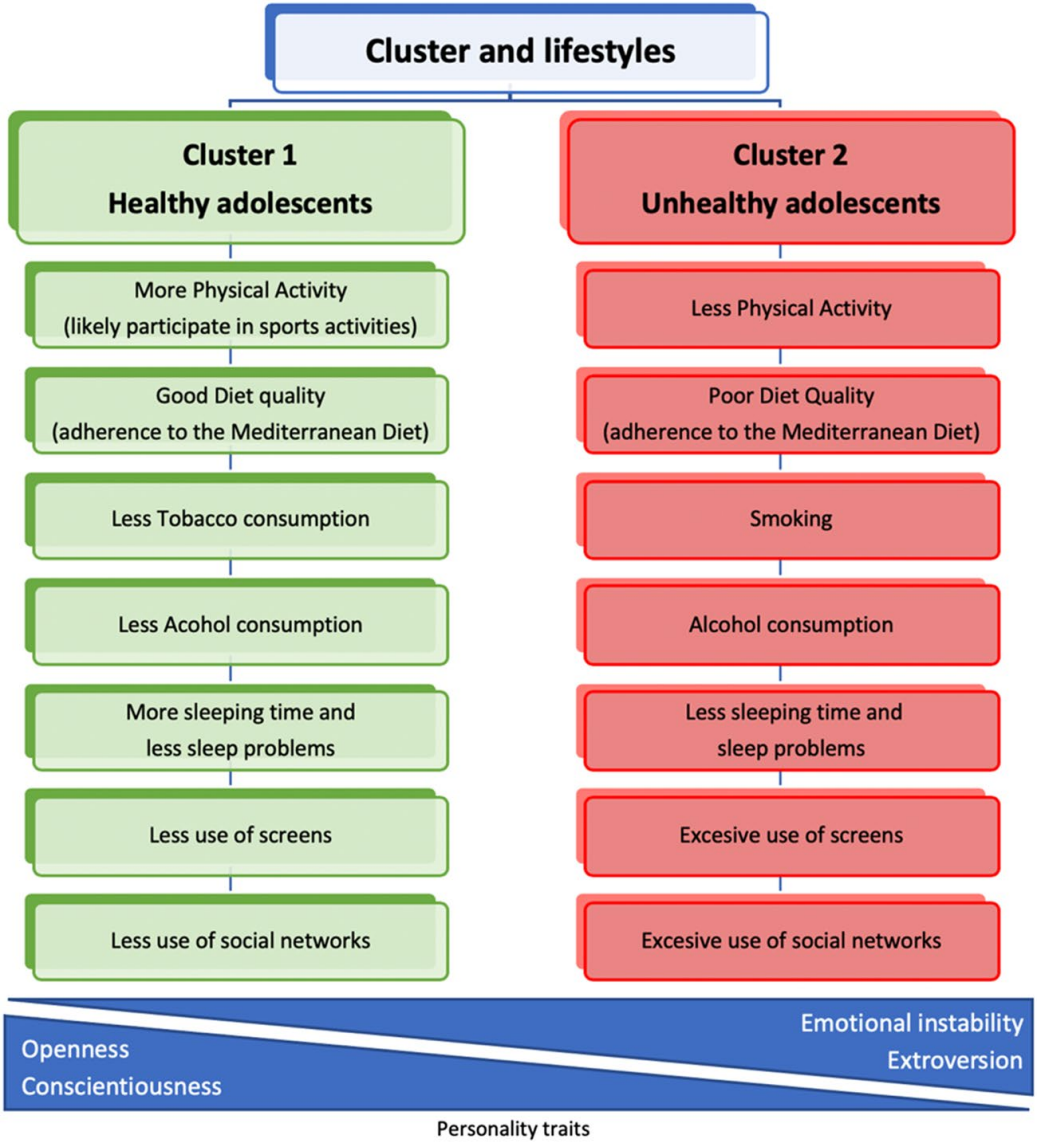
Clusters of adolescents’ health-related lifestyles and personality traits.

Our cluster analysis of the health-related lifestyles indicated the presence of two different clusters (Fig. 2). This analysis explained the grouping of all 695 adolescents included. We characterized these clusters as follows: Cluster 1 (Healthy lifestyles) was shaped by adolescents that were more likely to participate in sports activities, good adherence to the Mediterranean diet, less tobacco and alcohol consumption, less sleep problems, more sleeping time and less use of screens and social networks; and Cluster 2 (Unhealthy lifestyles) was shaped by adolescents were less physical activity, low adherence to the Mediterranean diet, smoking, weekly alcohol consumption, sleep problems and sleeping less hours and excessive use of screens and social networks.

There were significant differences between clusters according to gender (χ^2^_(1)_ = 6.447; p = 0.011) and parental education (χ^2^_(1)_ = 4.507; p = 0.034). Adolescents in Cluster 1 (healthy lifestyles) showed significantly higher scores of conscientiousness and openness and lower levels of extroversion and emotional instability. Adolescents in Cluster 2 (unhealthy lifestyles) showed higher scores of extroversion and emotional instability (Table 4). There was no statistically significant association with agreeableness.

**Table 4:** Characteristics of the clusters and association with personality (n = 695).

	Cluster 1 (n = 456, 65.6%) Healthy	Cluster 2 (n = 239, 34.4%) Unhealthy	p
Health-related lifestyles	n (%) or Mean (SD)		
Physical activity	0.08 (0.97)	−0.15 (1.01)	0.007
Mediterranean Diet adherence	0.29 (0.94)	−0.45 (0.88)	<0.001
Tobacco consumption	−0.18 (0.56)	0.26 (1.38)	<0.001
Alcohol consumption	−0.47 (0.76)	0.60 (0.93)	<0.001
Sleep problems	−0.17 (0.91)	0.23 (1.10)	<0.001
Sleep time	0.16 (0.90)	−0.27 (0.96)	<0.001
TV time	−0.22 (0.79)	0.29 (1.12)	<0.001
Screen time	−0.42 (0.50)	0.65 (1.07)	<0.001
Social Network time	−0.48 (0.46)	0.76 (1.14)	<0.001
Personality traits			
Conscientiousness	0.27 (0.95)	−0.12 (0.96)	<0.001
Openness	0.14 (0.98)	−0.07 (0.97)	0.011
Extraversion	−0.14 (1.00)	0.17 (0.90)	<0.001
Agreeableness	0.04 (0.97)	−0.05 (0.97)	0.960
Emotional instability	−1.26 (0.95)	0.10 (1.01)	0.002
Sociodemographic factors			
Gender			
Male	212 (46.5%)	86 (35.9%)	0.011
Parental education			
Secondary studies or higher	268 (58.8%)	110 (49.8%)	0.034

All cluster and personality variables were standardized (z-scores).

## Discussion

Our study provides important insights about acquisition of healthy lifestyles during adolescence and indicates that personality traits and parental education are associated with health-related lifestyle behaviours among adolescents after adjustment for age, sex and educational level. High conscientiousness scorers were more likely to adhere to the Mediterranean diet, smoked less, consumed less alcohol, and made better use of social networks. High extraversion scorers were more likely to engage in physical exercise; however, they were more likely to smoke and drink regularly, and adhered poorly to the Mediterranean diet. High scores in emotional instability were related to higher consumption of tobacco and alcohol, and sleep problems. The effect size of the association between personality traits and adherence to the Mediterranean diet and physical inactivity were small, however the association between extraversion (d = −0.460), emotional instability (d = −0,460) and smoking were of medium effect, and medium to large effect (d = −0.612) for emotional instability and sleeping problems. In addition, the educational level of the parents was also associated to diet and sleep problems.

A previous online survey in the United States found similar results^33,34^. High conscientiousness was associated with healthy behaviours, such as eating more fruits and vegetables, sleeping enough hours, and using the seat belt, whereas a high score in extraversion was associated with tobacco and alcohol consumption. Previous studies also showed an association between extraversion and increased consumption of tobacco and alcohol^35,36^.

Conscientiousness is defined as the tendency to be organised, in addition to complying with social norms, controlling impulses, being hardworking and responsible for obligations towards others^29^. According to a review^37^, the most important aspect of conscientiousness would be self-regulation that includes impulse control. Self-regulation is originated in childhood and adolescence, and is completely developed in the adult stage^38^. In our study, the association observed between conscientiousness and adherence to the Mediterranean diet could be mediated by the capacity for self-regulation. Responsible individuals would be able to delay the immediate gratification of less healthy diets and adhere to a healthy diet to minimise future health hazards.

In addition, personality traits could also influence the choice of situations or environment that would facilitate the adoption of healthy lifestyles^33^. Conscientiousness and healthy lifestyles would provide a positive feedback for maintaining high levels of conscientiousness; thus, personality and health behaviours may be reciprocally related across the lifespan^39^.

The present study indicated that extraversion would be associated with sports activities. The most extraverted students would search for additional external stimulation, and may be more willing to performance sports, but they are inherently social and could be also engaged in alcohol intake, tobacco consumption, or excessive use of social networks.

The recommended daily amount of sleep for an adolescent (aged 14 to 17 years) is between eight and ten hours^29,40^. However, we found that on school days most adolescents do not sleep eight hours, these results are in concordance with surveys from different countries^29,32^. Sleep deprivation increases with age^41^, although the importance that this finding may have for adolescents is still being discussed^31,42,43^. In our sample, low levels of parental education and emotional instability were associated with higher prevalence of sleep problems in adolescence. The association of parental education and adolescents sleep problems—which has not been previously reported in the literature—could be mediated by reduced ability to effectively protect sleep hygiene and emotional regulation of adolescents, and, perhaps, also their sociability, since the two-way interaction between sleep and social bond has also been described^30^. Sleep deprivation in adolescents is linked to screens and social networks use^44^. In our sample, on average, adolescents spent seven hours per day on screens (television, tables, computers, etc). This far exceeds the two hours recommendations of screen use among adolescents^40^, and most of this time was not devoted to school activities but to social networks (5.4 hours on average).

The results of cluster analysis showed that one of three adolescents had unhealthy behavior patterns. Adolescents health-related lifestyles should not be considered as isolated factors, because lifestyles tend to cluster (healthy and unhealthy lifestyles). Considering these results, health promotion interventions in adolescents should focus on multiple behaviors rather than any single behaviour^45^.

Our findings showed a similar associations, between personality traits and health-related lifestyles, to those reported among adult population^12,46,47^. Mean levels of conscientiousness increase across adolescence and there are individual differences in the degree and direction of these personality traits changes^8^. Different strategies for intervention focused on social-context or on increasing conscientiousness have been suggested. Although other actions focused on the behaviours associated with conscientiousness rather than on the underlaying personality trait may be more useful and cost efective^48^. Future studies should confirm if early psychoeducational interventions based on personality traits could prevent risk health behaviours to decrease health and mental disorders in adulthood.

### Strengths and limitations

The present study has some strengths and limitations. First, the measures of health-related lifestyles were self-reported by the adolescents, therefore, we cannot rule out complacency biases, especially in students with higher levels of pleasantness. Secondly, some of the outcome variables could be influenced by parents and the family environment, which could reduce the individual effect of personality traits. Although there is also a shared family component on personality in terms of genetic and environmental influences^49^. Physical activity, sleep hours, and screen hours were collected through short ad hoc questionnaires, however relevant information on personality and adherence to the Mediterranean diet were collected through validated questionnaires in the Spanish population with good psychometric characteristics. Study strengths included the longitudinal design, large sample size and the evaluation of different lifestyles at the same time.

## Conclusions

Conscientiousness, extraversion, emotional instability and parental education are independent factors associated with the acquisition of adolescent healthy lifestyles, these findings have important implications for healthy lifestyles promotion among adolescents. Individuals with high extraversion levels are generally active. These results underpin the importance to provide and promote healthy activities among students, such as social and sports activities. In addition, personality traits can help identifying high-risk groups and perform appropriate interventions based on their characteristics. Finally, the confirmation of the importance of parents and socio-economic status in the acquisition of healthy lifestyles during adolescence. Our results indicated that adolescents whose parents had lower educational level were more likely to eat unhealthy food and report sleep problems. Any school-based intervention for the promotion of health should consider the influences of the social determinants of health and their perpetuation between generations to reduce health inequities.

## Methods

### Participant recruitment

The participants of the present study were adolescents, aged 14 to 15 years, who participated in the ITACA study^50,51^. The initial sample size of the cohort study included 1,708 secondary students (aged 11 to 12 years) from 16 Spanish secondary schools. The schools were randomly assigned to a multifactorial intervention to reduce the prevalence of smoking or as a control group. The data presented include the second and the third evaluation. In the second evaluation (2014–2015), we collected data relating to sociodemographic characteristics, level of parents’ education, and personality. In the third evaluation (May-June 2016), we collected data on the main lifestyles related to health. This evaluation was completed by 1,123 students from 12 schools, 824 of which had data in both the second and the third evaluation. Finally, there were 695 students with valid data for this analysis.

### Procedures and measures

The data were self-reported in the classrooms on a school day, during one of the lessons. The students of the third year of secondary education responded to the survey in 60 minutes (second evaluation), and the students of the fourth year in 45 minutes (third evaluation). The surveys were administered by two previously trained interviewers. The teachers were asked to leave the classrooms to ensure the confidentiality of the students. The students who participated and theirs fathers or mothers signed an informed consent form. The study protocol was approved by the Institutional Review Board of the Balearic Islands Health Service (CEI-IB Ref. No. 1146/09). The study was conducted according to the ethical guidelines of the Declaration of Helsinki.

### Personality traits

Personality traits were assessed using the Big Five Questionnaire for Children and Adolescents (BFQ-CA)^52^ designed to assess the five basic personality traits, namely: extraversion, agreeableness, conscientiousness, emotional instability and openness. Extraversion assesses characteristics such as enthusiasm, assertiveness, and self-esteem. Agreeableness assesses sensitivity and concern for others and their needs. Conscientiousness evaluates compliance with commitments and order. Emotional instability refers to feelings such as anxiety, depression, dissatisfaction, and anger. Finally, openness refers to self-reported intellectual capacity -especially in the school domain- cultural interests, fantasy, and creativity^52^. The Spanish adaptation of the questionnaire included 65 items with five possible answers^53^. The reliability of this questionnaire, measured by Cronbach’s alpha, was high for the different traits: 0.77 for extraversion; 0.71 for agreeableness; 0.87 for conscientiousness; 0.77 for emotional instability and 0.82 for opening. Previous studies have already demonstrated good psychometric properties of the BFQ-CA^54^.

### Parental education

The measurement of parental education describes their highest educational level. The categories were as follows: (a) incomplete primary education (less than six years); (b) complete primary education (six to eight years); (c) secondary education (four to six years); and (d) university degree^50,55^. The highest parental education variable was established by taking the highest educational level obtained by either parent. These two variables were transformed into a new variable that contained the highest value achieved by one or both parents, classified into two categories: (1) primary studies, compulsory studies, or lower; and (2) secondary studies, higher degree or university professional training.

### Mediterranean Diet

The Mediterranean Diet Quality Index was determined using the KIDMED test^56^. The KIDMED index included 16 questions about food habits, and classified individuals according to the scores of the Mediterranean Diet Quality index differentiated into three categories: high adherence, optimal Mediterranean diet (8 and 12 points); medium adherence, need for improvement in the food pattern to adapt it to the Mediterranean model (4 and 7 points); and low adherence, very low quality of the diet (0 and 3 points).

### Physical activity

The level of weekly physical exercise was assessed with a question about the number of days per week they performed some sport extra-curricularly: (1) never; (2) once a week; (3) twice a week; (4) three times a week; (5) four times a week; and (6) five or more times a week. This variable was categorised as performing weekly physical exercise (yes/no).

### Tobacco

Smoking was assessed using seven items adapted from a previously validated questionnaire^50^, which assessed the consumption of tobacco categorised as weekly tobacco consumption (yes/no).

### Alcohol consumption

Alcohol consumption was quantified using the following question: How often do you drink alcoholic beverages? = (1) never-almost never; (2) sometimes a month; (3) on weekends; and (4) daily or almost daily. This variable was categorised as weekly consumption of alcoholic beverages (yes/no).

### Sleep problems

Sleep problems were assessed with the following question: Do you have sleep problems? = (1) never-almost never; (2) sometimes a month; (3) many nights (one or more nights a week); and (4) every night. This variable (yes/no) was categorised according to having sleep problems one or more times per week. In addition, the number of hours they slept on school days and on weekends was recorded.

### Screen time and use of social networks

Television, screen, and social media hours were collected separately for school days and weekends. The variable was categorised as excessive use of screens (yes/no) and/or social networks (yes/no) if more than two hours per day were used on school days.

### Statistical analyses

The listwise deletion method (with complete case deletion) was used to account for missing data^57^. Quantitative variables were expressed as mean and standard deviation, and qualitative variables in the form of absolute frequencies and percentages. The analysis of the differences between groups for the quantitative parameters was performed using Student’s *t*-test. Pearson’s chi-square test was used to assess the differences between the qualitative variables. Listwise deletion method (complete case) were used for handling missing data.

The effect of personality on healthy lifestyle habits was assessed by standardising the values of different personality traits and adjusting different binary logistic regression models in which the dependent variables were: (1) low adherence to the Mediterranean diet (KIDMED < 7); (2) not performing physical exercise at least once a week; (3) weekly tobacco consumption; (4) weekly alcohol consumption; (5) having weekly sleep problems; (6) excessive use of screens; and (7) excessive use of social networks.

A general linear model was also adjusted. The dependent variable was adherence to the Mediterranean diet as a continuous variable, and the independent variables were the levels of conscientiousness, parental education, and age and sex of the adolescents.

Cluster analysis (k-means algorithm) was used to identify groups of students who shared common health-related lifestyles. This technique identifies subgroups of cases in adolescents based on shared characteristics. All variables were standardized before the cluster analysis. This method identifies uniform groups by maximizing inter-group variance and minimizing intra-group variance^58^. Subsequent analyses (t-test and Chi-Square) were used to identify differences between the clusters and sociodemographic characteristics (gender and parental education) and personality traits.

It was considered that there were statistically significant differences when the *p*-value was <0.05. All data were analysed using the SPSS Statistics 24 software (IBM Company, Chicago, Illinois, United States).
